# Assessment of *Leishmania infantum* infection in equine populations in a canine visceral leishmaniosis transmission area

**DOI:** 10.1186/s12917-019-2108-1

**Published:** 2019-10-30

**Authors:** Taiane Acunha Escobar, Gabriela Dowich, Thália Pacheco dos Santos, Luísa Zuravski, Claudia Acosta Duarte, Irina Lübeck, Vanusa Manfredini

**Affiliations:** 1 Biochemistry Postgraduate Program, Federal University of Pampa, 118 BR 472, Uruguaiana, Rio Grande do Sul Km 592 Brazil; 2 Animal Science, Postgraduate, Federal University of Pampa, 118 BR 472, Uruguaiana, Rio Grande do Sul Km 592 Brazil; 3 Pharmacy, Federal University of Pampa, 118 BR 472, Uruguaiana, Rio Grande do Sul Km 592 Brazil; 4 Veterinary Medicine, Federal University of Pampa, 118, BR 472, Uruguaiana, Rio Grande do Sul Km 592 Brazil

**Keywords:** PCR, Diagnosis, Clinical valuation, Haematology, Biochemical analysis, Horse, Dog

## Abstract

**Background:**

Leishmaniosis, zoonosis that produces significant public health impacts, is caused by *Leishmania infantum*. Canines are the main domestic reservoir and, besides humans, other species of mammals could be infected when living in endemic areas. In this study, we detected equine *Leishmania infantum* infections in a canine visceral leishmaniosis transmission area and evaluated the clinical, haematological, biochemical and oxidative stress disorders. This study was conducted in Uruguaiana, Rio Grande do Sul, south of Brazil. Peripheral blood samples were collected from 124 animals (98 horses and 26 dogs) of both genders and several breeds after they underwent general and dermatologic examinations.

**Results:**

Twenty five *Leishmania infantum* infected animals (20.16%), 14 horses and 11 dogs were detected by PCR (Polymerase Chain Reaction) amplification of kinetoplast DNA regions with 96% homology to *Leishmania infantum* (GenBank Accession No. L 19877.1). The clinical and haematological alterations of infected equines were skin lesions, nodules, lymphadenopathy, decreased levels in red blood cells and haematocrit (*p* < 0.05) and increase in urea serum concentration (*p* < 0.05), while CVL presented a decrease in red blood cells counts (*p* < 0.05), increase in lymphocytes (*p* < 0.05), and decrease in neutrophil-lymphocyte ratio (*p* < 0.05). Oxidative stress markers of plasma protein carbonyl and plasma lipid peroxidation were not statistically significant (*p* > 0.05) in both species.

**Conclusions:**

To our knowledge, this has been the first leishmaniosis equine survey performed in south of Brazil, caused by *Leishmania infantum* that were able to initially identify haematological and biochemical changes in the species, even in asymptomatic animals. We present evidence supporting those findings of haematological and biochemical changes could be related to infection. Surprisingly, the clinical manifestations of equine infection were similar to those found in canine visceral leishmaniosis. The equine population could be play an important role in the cycle of leishmaniosis in south Brazil and consequently indicates a great risk of public health. This evaluation of infected animals is important to establish the clinical and laboratory parameters involved in the disease progression.

## Background

Leishmaniosis is a zoonotic disease distributed globally, capable of causing significant impacts on public health. It has high lethality if untreated, as well as impossibility of vector eradication and consequently, a proven trend of expansion [1]. Visceral leishmaniosis (VL) is caused by *Leishmania infantum* protozoa and affects humans, as well as domestic dogs, which are the main reservoir of this infection [2]. Currently, the disease is widespread throughout Brazil. The disease was not endemic in Rio Grande do Sul until 2008 when the first canine case was diagnosed. Since then, seven municipalities on the cross border with Argentina, neighbouring country, including São Borja and Uruguaiana cities were transmission areas for CVL with the vector *Lutzomyia longipalpis* has been reported [3, 4].

In view of epidemiological situation at the western border of the state of Rio Grande do Sul, the identification of possible host species of leishmaniosis is a challenging and necessary task, since it is known that besides dogs, dozens of wild and domestic mammals, including equines, have already been identified to carry the infection in other regions [5–7]. The challenge is due to the variability of clinical signs in animals, in addition to the large number of asymptomatic individuals. In China, asymptomatic sheep infected with *Leishmania infantum* were suggested to contribute to VL transmission [8].

Evidences of horses residing in endemic areas for canine visceral leishmaniosis (CVL) and human visceral leishmaniosis (HVL) were susceptible to become infected with *Leishmania infantum*, only reinforce the importance of researching species that act as a food source for vectors or participants in the epidemiological chain [9]. Equine infection by the protozoan *Leishmania infantum* has been reported in South America and Europe [6, 10–12]. Unlike standardized human and canine diagnosis, parasitological, molecular and serological tests are used for the detection in other species, added to the clinical evaluation of the individual [6, 13, 14]. Besides, the disease in horses, can cause clinical manifestations (nodules, skin lesions), it can be spontaneously cured, or the host immune system can developing different mechanisms to evade or modulate the immune response keeping the host asymptomatic and hindering initial clinical evaluation and suspicion of infection [11, 12, 15, 16].

Haematological and biochemical markers of CVL have been identified [17, 18] however, it remains unclear at what extent the occurrence of clinical signs and variations in laboratory parameters can occur in horses. In this way, complementary tests can be used associated with diagnostic techniques, in order to secure more accurate diagnosis of the disease while providing a view of the individual’s condition at the moment of collection.

In Rio Grande do Sul state, south of Brazil, the western border region, the use of draft horses for agricultural, farming and transportation activities are common. In this transmission area for CVL, horses living in close contact with humans and dogs, especially in the urban area, can participate in the transmission cycle through interaction with vectors. Because, they are excellent attractions and blood supply for the feeding of sand flies, thus stimulating the proliferation of these vectors [19]. There have been reports of infection in horses from endemic areas in other regions of the world, presumably because they are in contact with domestic reservoirs and vectors [13, 16, 20].

*Leishmania infantum* is not only pathogenic for animals, causes a serious disease in humans too, for this reason, its important pay attention for the occurrence of infection in potential reservoirs, especially in CVL endemic and transmission areas. The incidence rates of leishmaniosis in the human and canine population in the Rio Grande do Sul western border evidences the urgent need to investigate the infection in horses. Due to the lack of information, that guided the research, the aims were evaluating the presence of clinical signs, haematological, biochemical and oxidative stress disorders that may be present in equines infected by *Leishmania infantum* in a CVL transmission area.

## Results

This study evaluated the clinical signs, haematological, biochemical and oxidative stress of 124 animals (98 horses and 26 dogs) of both genders and several breeds, from a CVL transmission area in the south of Brazil. The animals tested in this experiment, lived in areas with large amounts of organic matter, such as landfill sites, where there were substrates necessary for the multiplication of leishmaniosis vector. The draft horses generally slept in the backyards of residences, belonging to socio-economically deprived communities in semi-urban areas. Owners of 30 horses reported some previous diseases or clinical signs, the main complaints were: alopecia in different regions of the body and nasal discharge. One animal had ataxia and another had torticollis, four females were pregnant at the time of collection.

The PCR results, of kinetoplast DNA gene markers, revealing a detection of *Leishmania* amplicons in 20.16% samples. The direct sequencing fragments performed to confirm the identity of protozoan species in these samples showed 96% homology to the *Leishmania infantum* (GenBank Accession No. AF103741.1). The percent of *Leishmania infantum* infected horses were 14.3%, 14 animals among 98 of total equine population of the study. The molecular diagnosis of CVL showed 42.3% of infected dogs (11 animals) living in the same areas of infected horses.

Equine population was composed by 43 males (aged 0.5–24 years) and 55 females (aged 0.5–27 years). According to sex, the female *Leishmania infantum* infected equines were 64.3%, whereas the male rate *Leishmania infantum* infected equines was 35.7%. In the canine population, compound by 13 males and 13 females, the highest prevalence of CVL were recorded in females with 81.8% (*p* < 0.05).

Five infected equines were symptomatic (SyE), the clinical alterations were skin lesions (*n* = 3), nodules (*n* = 1), lymphadenopathy (n = 1) (see Table 1) and nine infected animals were without of clinical signs (AE). In CVL, five infected dogs were SyD and six were AD, in addition, the SyD group presented conjunctivitis. The clinical signs frequency of *Leishmania infantum* infected and control groups did not shows differences, most of evaluated animals were not dehydrated (Table 1).

**Table 1:** Clinical signs frequency of control group and *Leishmania infantum* infected group by Chi-square test

	Control Group	*Leishmania infantum* Infected Group	*p- value*
	(N/%)	(N/%)	
Equine Samples Parameters			
Low Body mass (*n =* 46)	14 (30.43)	3 (6.52)	1.00
Skin lesions (*n =* 98)	15 (15.30)	3 (3.06)	1.00
Lymphadenopathy (*n =* 98)	2 (2.04)	1 (1.02)	0.90
Dehydration (*n =* 46)	4 (8.69)	0 (0)	0.97
Eyes pale mucous membrane (*n =* 55)	13 (23.63)	1 (1.81)	0.40
Oral pale mucous membrane (*n =* 45)	2 (4.44)	0 (0)	1.00
Canine Samples Parameters			
Low Body mass (*n =* 25)	5 (20)	5 (20)	0.67
Skin lesions (*n =* 26)	3 (11.53)	2 (7.69)	1.00
Eyes pale mucous membrane (*n =* 24)	6 (25)	3 (12.50)	1.00

Due to the lack of adequate structure for the animal’s containment, in some occasions, the clinical evaluation and parametric indices were performed on as many animals as possible (Table 1), as well as to some laboratory analyses due to the obtaining of little biological material at the time of collection.

The parametric indices of equine infected group show an increase of HR (heart rate) while in the canine specie the only change was body temperature (Table 2).

**Table 2:** Comparison between parametric indices of control group and *Leishmania infantum* infected equines and canines

	Control Group	*Leishmania infantum* Infected Group	*p- value*
	(X±SD)	(X±SD)	
Equine Samples Parameters			
CRT (*n =* 83)	1.89 ± 0.43	2.00 ± 0.00	0.36 ^b^
RR (*n =* 94)	21.59 ± 10.86	18.15 ± 5.50	0.30 ^b^
HR (*n =* 95)	44.10± 13.74	49.23 ± 9.67	0.05 ^b^*
BT (*n =* 81)	36.97 ± 0.95	37.06 ± 0.76	0.74 ^a^
Canine Samples Parameters			
CRT (*n =* 16)	1.75 ± 0.46	1.88 ± 0.64	0.69 ^b^
RR (*n =* 24)	35.20 ± 33.94	28.44 ± 9.26	0.85 ^b^
HR (*n =* 25)	102.40 ± 34.4 6	102.40 ± 20.93	1.00 ^a^
BT (*n =* 23)	38.73 ± 0.39	38.35 ± 0.23	0.00 ^b^*

*X* Mean, *SD* Standard Deviation; *The difference was considered significant when *P* value less than 0.05; ^a^ Student T test; ^b^ Wilcoxon test; *CRT* Capillary Refill Time, *RR* Respiratory Rate, *HR* Heart Rate, *BT* Body Temperature

Results from equine haematological analysis, in see Table 3, revealed that the number of RBC and HCT were shorter in infected group than the CE group (*p* < 0.05). While in both groups MCV and MCHC values were acceptable. The granulocyte absolute counts (eosinophil and basophil) and monocytes values were acceptable in both groups, although neutrophil showed an decrease values in *Leishmania infantum* infected group (*p* < 0.05) and relative basophils had an increase in this group (*p* < 0.05). The same change in RBC rates was observed in the canine specie (*p* < 0.05). The PLR values were shorter (*p* < 0.05) and the lymphocytes (absolute and relative) were higher in (*p* < 0.05) canine infected group (see Table 3).

**Table 3:** Haematological profile in control group and *Leishmania infantum* infected equines and canines

	Control Group	Infected Group	*p- value*	Control Group	Infected Group	*p- value*
	(X±SD)	(X±SD)		(X±SD)	(X±SD)	
	Equine Samples			Canine Samples		
RBC (x10^6^ cells/μL)	7.51 ±1.26	6.69 ± 1.58	0.03 ^a^*	5.75 ± 0.98	4.90 ± 0.81	0.02^a^*
HCT (%)	36.76 ± 5.52	32.47 ± 7.11	0.01 ^a^*	38.47 ± 7.02	33.38 ± 6.03	0.06 ^a^
Hb (g/dl)	12.48 ± 1.81	11.21 ± 2.32	0.09 ^b^	13.00 ± 2.86	11.33 ± 2.20	0.12 ^a^
MCV (fl)	49.17 ± 3.35	49.10 ± 5.01	0.86 ^a^	66.84 ± 3.71	67.99 ± 4.07	0.37 ^b^
MCHC (g/dl)	34.01 ±1.27	34.69 ± 1.30	0.06^b^	33.60 ± 2.03	33.90 ± 1.34	0.24 ^a^
PLT (x10^3^ cells/μL)	163.51 ± 119.58	145.50 ± 72.07	0.81^b^	169.30 ± 101.58	186.45 ± 139.86	0.78 ^a^
WBC (x10^3^ cells/μL)	11.88 ± 3.27	11.23 ±3.87	0.36 ^a^	13.87 ± 5.00	18.57 ± 8.22	0.83 ^a^
Absolute Neutrophils Rods (cells/μL)	340.48± 353.79	288.43 ± 229.50	0.72 ^b^	1.764 ± 2.205	2.231 ± 1.220	0.09 ^a^
Relative Neutrophils Rods (%)	2.99 ± 3.07	2.71 ± 2.40	0.99 ^b^	11.73 ± 10.97	13.18 ± 7.44	0.30 ^a^
Absolute Neutrophils (cells/μL)	6.118 ± 2.171	4.774 ± 1.598	0.01 ^a^*	7.370 ± 2.972	8.768 ± 5.270	0.44 ^a^
Relative Neutrophils (%)	51.61 ± 10.42	45.29 ± 14.24	0.11 ^b^	54.47 ± 18.18	44.27 ± 12.15	0.12 ^a^
Absolute Eosinophils (cells/μL)	460.75 ± 319.31	433.07 ± 372.51	0.55 ^b^	395.33 ± 499.66	511.82 ± 687.42	0.73 ^b^
Relative Eosinophils (%)	4.14 ± 3.08	4.07 ± 3.47	0.83 ^b^	2.80 ± 3.52	2.64 ± 2.76	0.81 ^b^
Absolute Basophils (cells/μL)	95.61 ± 137.59	148.64 ± 161.38	0.09 ^b^	96.80 ± 195.41	126.45 ± 170.93	0.49 ^b^
Relative Basophils (%)	0.80 ± 1.07	1.21 ± 0.89	0.05 ^b^*	0.80 ± 1.82	1.00 ± 1.41	0.47 ^b^
Absolute Lymphocytes (cells/μL)	4.444 ± 1.969	5.290 ± 3.543	0.42 ^a^	3.148 ± 2.093	5.728 ± 2.531	0.00 ^a^*
Relative Lymphocytes (%)	36.99 ± 10.78	44.36 ± 14.48	0.08 ^b^	22.20 ± 10.97	33.36 ±12.16	0.02 ^a^*
Absolute Monocytes (cells/μL)	410.63 ± 366.48	300.71 ± 316.82	0.20 ^b^	1.097 ± 909.43	1.207 ± 1.381	0.81 ^a^
Relative Monocytes (%)	3.31 ± 2.66	2.36 ± 2.13	0.24 ^b^	13.33 ± 21.77	5.55 ± 4.54	0.20 ^a^
PLR	0.04 ± 0.03	0.03 ± 0.02	0.62 ^b^	0.08 ± 0.11	0.03 ± 0.02	0.04^b^*
NLR	1.68 ± 0.79	1.30 ± 0.78	0.10 ^a^	4.78 ± 5.22	2.09 ±1.13	0.06 ^b^

*X* Mean, *SD* Standard Deviation; *The difference was considered significant when *P* value less than 0.05; ^a^ Student T test; ^b^ Wilcoxon test; Reference values for equine specie: RBC: 6,5-12,5; HCT: 32-52; Hb: 11-19; MCV: 34-58; MCHC: 31-37; PLT: 100-600; WBC: 5.5-12.5; Neutrophils Rods: 0-100; Neutrophils: 2.700-6.700; Eosinophil: 0-925; Basophil: 0-170; Lymphocytes: 1.500-5.500; Monocytes: 0-800; Reference values for canine specie: RBC: 5,5-8,5; HCT: 37-50; Hb: 12-18; MCV: 60-67; MCHC: 32-37; PLT: 200-500; WBC: 6-17; Neutrophils Rods: 0-300; Neutrophils: 3.000-11.500; Eosinophil: 150-1.250; Basophil: 0-200; Lymphocytes: 1.000-4.800; Monocytes: 150-1.350

The equine biochemical profile results of urea serum concentration were significantly increasing (*p* < 0.05) in *Leishmania infantum* infected group. This biochemical disorder was the only one found in the infected group. Mean values of hepatic enzymes (AST, GGT) were in agreement with the reference values for the specie and total protein in both groups were higher than reference limits, although there was no statistical difference in the comparison between them (see Table 4). In the canine species we did not found significant changes in biochemical parameters in infected animals (see Table 4 ).

**Table 4:** Biochemical profile in control group and *Leishmania infantum* infected equines and canines

	Control Group	Infected Group	*p- value*	Control Group	Infected Group	*p- value*
	(X±SD)	(X±SD)		(X±SD)	(X±SD)	
	Equine Samples			Canine Samples		
AST (Ul/L)	243.60 ± 129.49	218.64 ± 46.41	0.55^b^	7.83 ± 3.86	12.71 ± 9.19	0.28^a^
GGT (Ul/L)	20.77 ± 12.49	15.91 ± 4.94	0.30 ^b^	5.57 ± 2.07	6.17 ± 3.06	0.68 ^a^
Creatinine (mg/dL)	1.20 ± 0.26	1.22 ± 0.31	0.96 ^b^	1.14 ± 0.25	1.15 ± 0.53	0.95 ^a^
Urea (mg/dL)	48.31 ± 11.46	57.52 ± 13.61	0.01^a^*	24.00 ± 2.12	28.04 ± 19.90	0.96 ^a^
Total Protein (g/dl)	9.82 ± 0.99	9.35 ± 0.97	0.14 ^a^	6.55 ± 0.94	7.16 ± 0.99	0.22 ^a^
CK (Ul/L)	387.85 ± 212.38	365.91 ± 171.44	0.87 ^b^	**-**	**-**	
ALB (g/dl)	4.08 ± 0.49	4.06 ± 0.50	0.91^a^	2.55 ± 0.50	2.27 ± 0.37	0.23 ^a^
Globulin (g/dl)	5.73 ± 1.21	5.29 ± 1.16	0.28 ^b^	3.99 ± 1.31	4.98 ± 1.29	0.16 ^a^
Gamma Globulin	18.81 ± 3.81	19.35 ± 4.89	0.67 ^a^	1.15 ± 0.41	1.61 ± 0.92	0.31^a^
A/G	0.75 ± 0.22	0.63 ± 0.22	0.29 ^a^	0.75 ± 0.42	0.49 ± 0.18	0.17 ^a^

*X* Mean, *SD* Standard Deviation; *The difference was considered significant when *P* value less than 0.05; ^a^ Student T test; ^b^ Wilcoxon test. Reference values for equine specie; AST: 120-480; GGT: 10-62; Creatinine: 0.96-1.3; Urea: 21.6-51; Total Protein: 5.8-8.7; CK: 40-280; ALB: 2.6 – 3.8; Globulin: 2.5 – 4.1; Gglobulin: No reference values were found in the literature. A/G: 0.5-1.7; Reference values for canine specie: AST: 23-66; GGT: 1.2-6.4; Creatinine: 0.5-1.5; Urea: 15-65; Total Protein: 5.4-7.1; ALB: 2.6 – 3.3; Globulin: 2.5 – 4.1; Gglobulin: 0.8-1.8; A/G: 0.5-1.7

**Fig. 1:**
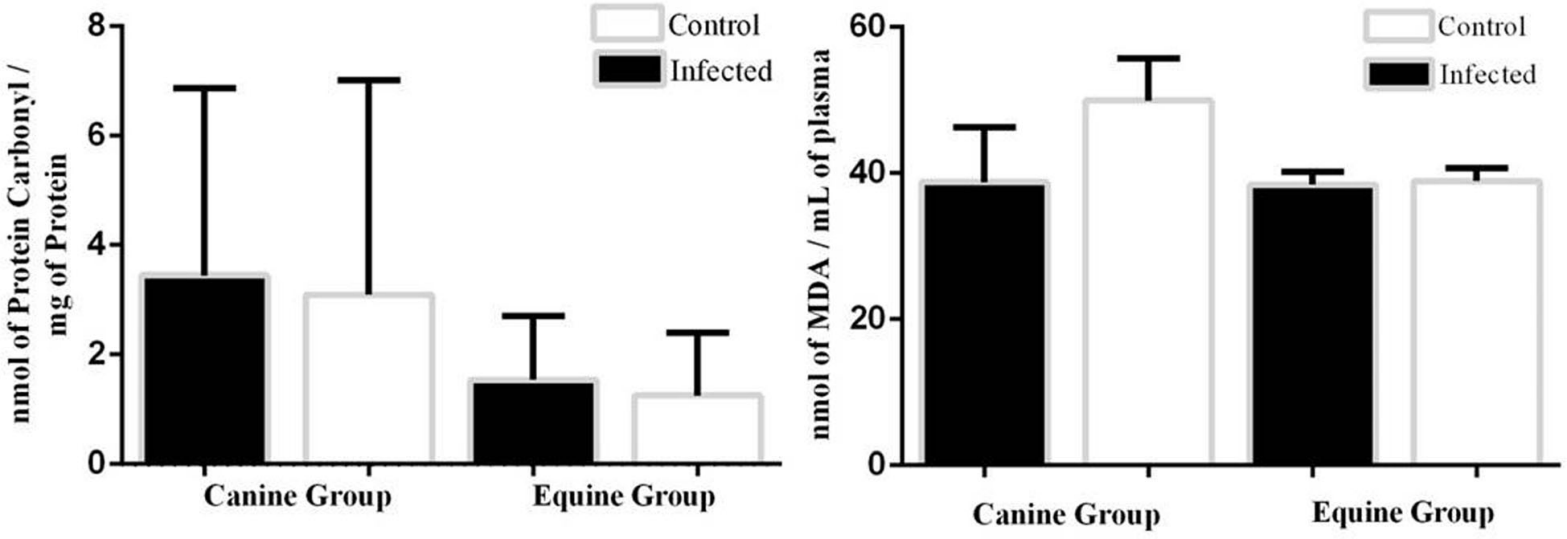
Markers of oxidative stress in canine and equine groups. Means ± SE in control and infected animals. 1A: plasma protein carbonyl; 1B: plasma lipid peroxidation (TBARS)

The results reported of means ± SD for oxidative stress markers carbonyl levels and TBARS of were not statistically significant (*p* > 0.05) in both equine and canine species (Fig. 1).

## Discussion

The study revealed *Leishmania infantum* infection in the equine species of Uruguaiana municipality’s semi-urban regions as a new transmission CVL area in the south of Brazil where they were exposed to the vector and mainly inhabiting the same places as infected canines.

These results agreed with findings of reported infected horses in different endemic areas around the world, including southeast Brazil [12]. Confirmation of *Leishmania infantum* equine infection in this region showed the urgency and importance of investigating this animal species, taking them into account as a mean of transportation (people and cargo) that circulates throughout the city at any given time of the day. From these findings we must evaluate the equines’ interaction with the vector species, even the degree of involvement of equines in the transmission to other hosts which has yet not been defined as a possible reservoir. They can also attract the vectors [16] and might guarantee their maintenance through the females feeding, contributing to the increasing incidence of *Leishmania infantum*. On the other hand, dogs, confirmed as domestic reservoirs, usually remain restricted to smaller regions of town. They do not cross the urban and semi-urban perimeter like the horses.

The CVL clinically displays a wide range of nonspecific symptoms, including skin lesions, which are present in most clinical cases [6], apathy, weight loss, enlarged lymph nodes, hepatomegaly, splenomegaly, and others [21]. Tegument and ocular signs, such as alopecia, hypotrichosis, exfoliate dermatitis, cutaneous and mucocutaneous ulcers, crusted scars, and onychogryphosis were recorded too [22]. We evaluated horses in an attempt to identify changes similar to those found in CVL. The cases described here, showed that the majority of the infected equines were without clinical signs, although SyE infected animals presented some apparent signs such as: skin lesions, nodules and lymphadenopathy, similar to clinical signs detected in dogs from the same areas. The clinical signs of leishmaniosis caused by *L. infantum* in horses had been described like temporary signs, which included nodules of variable size, in sometimes covered by eroded or ulcerated epidermis [10, 12, 15]. Healthy infected equines were reported in north Portugal and Spain [20, 23]. The absence of clinical changes in equine infection were related to a possible immunological ability to reduce parasitic load or which may be associated with this pattern of temporary signs that the animals present [6, 19]. Prevalence of equine subclinical *Leishmania infantum* infection, in endemic areas, is considerably higher than that the disease, as observed in other animals, cats and dogs [23, 24]. The ratios of symptomatic and asymptomatic dogs were similar, which requires a careful attention because it is a transmission area and little is studied about the dynamics of the disease in reservoirs and possible hosts, especially in regions, such as the one in the study, which dogs have close contact with horses.

Assessments of clinical and physical examination did not show signs and alterations in the infected animals except for HR that increased in infected group, but cannot be attributed only to *Leishmania infantum* infection, due to the physiological interference caused by the effort in this index which may possibly have occurred due to the short time interval between waiting for clinical examination or even the activities performed by these animals. Studies of infection in horses reported only the clinical changes caused by lesions, nodules and ulcers [12, 14, 19]. Alterations in parametric indices were not reported yet, so with this first finding of cardiac abnormality, we need to investigate the infected group to see if this change can be predictive of infection. Contrariwise, the evaluated dogs did not present this alteration.

Although several influencing factors in these animals, such as nutritional deficiency, stress due to heavy work, precarious conditions of animal health, it was possible evidence some indicative changes of the infection in haematological and biochemical tests. RBC and HCT decreased in equine infected group, as has been previously described in equine piroplasmosis, another disease caused by two protozoans (*Theileria equi* and *Babesia caballi*) in Egypt populations [25]. Perhaps protozoan’s infection in equines may present the same pattern of haematological disorders. Similarly, the canine infected group presented anaemia. In CVL, Alterations in laboratory parameters, particularly haematological, often reveal normocytic anaemia, thrombocytopenia [26].

A significant reduction in neutrophils was observed in equine infected group concomitant with uraemia. Neutrophils are the main effector cells in mammalian innate immunity; parasites like *Leishmania infantum* can modify the oxidative metabolism and causes apoptosis of neutrophils according to disease stage. In very severe stage of CVL, were observed decrease superoxide production and increased apoptosis associated with uraemia [27]. Because little is known about haematological changes in equine infection, it is not possible infer these findings at a severe stage of the infection, like in dogs. However, there are indications that at this stage in which animals were investigated, apoptosis of neutrophils could explain these reductions.

In humans, uraemia increases the rate of spontaneous neutrophil apoptosis [28], as well, the higher rate of spontaneous apoptosis and lower spontaneous viability of neutrophils in uremic groups are similar to previous studies in dogs [29, 30], similar to changes that we found in horses.

Deranged haematological and biochemical parameters is a feature of VL in humans [31], that can be used as an aid in HVL diagnosis and treatment. In CVL has found haematological, biochemical and oxidative markers, as well [29, 32]. In addition, the spread of infection in other species requires detailed scientific investigation to know the dynamics of the disease. We started with this study the identification of these alterations in equines to find establish prognostic biomarkers that could facilitate diagnosis, prophylactic and therapeutic procedures, since as in the other mammalian species, in the equines also there is a great variation of clinical signs and the possibility of not presenting any sign either.

Oxidative stress has been attributed to several parasitic diseases in the canine species, including infections caused by *Leishmania infantum* [29, 32]. It was not possible identify changes in oxidative stress markers levels in analysed groups, however, it’s a clear limitation of the present study, because these parameters are difficult to assess when it comes to samples from uncontrolled individuals, and especially when they are under adverse conditions of nutritional, hydration and physiological status.

As expected, was found a higher level of infection in dogs, in comparison with equine specie, since the region is a potential CVL transmission area and the main reservoir was in contact with vectors and other mammals. In Paraná state, Brazil, another study showed a similar rate of *Leishmania braziliensis* (16.6%) infection in horses by PCR diagnosis [13]. The characterization of populations of infected dogs is important to identify the main parameters associated with the development of VL [17, 33], attending as an initial parameters for the investigation of the VL development in other mammalian species.

Results of sequencing kDNA regions indicate the presence of *Leishmania infantum*, in Blast search, the sequences finds regions of similarity between biological sequences of *Leishmania infantum* infecting horses and dogs in this CVL transmission area. The infection of *L. infantum* was been reported in Europe [10, 11, 14, 15], in Brazil, the infection in horses have been identified as well [12]. The European [14, 15, 20, 23] and Brazilian reports of VL infection in horses in are restricted to serological and molecular evaluation, without evidences of haematological, biochemical and oxidative stress changes in this specie [12, 16].

## Conclusions

Our results showed the occurrence of infection by *Leishmania infantum* through molecular diagnosis in 14.3% equines and in 42.5% canines. Surprisingly, the clinical manifestations of equine infection were similar to those found in CVL. We present evidence supporting those values of HCT, RCB, neutrophil and urea changes in the equine species that could be related to infection. These results may aid in the identification of infected horses in endemic areas or in the transmission of CVL. It is suggested that the infected animals have a clinical follow-up during as well as the control of parasite load to assess whether haematological changes and identify which lymphocytes are acting on the immune response of the infection in order to relate to the absence of clinical signs. This evaluation of infected animals is important to establish the clinical and laboratory parameters involved in the evolution of the disease. To our knowledge, this has been the first leishmaniosis equine survey performed in south Brazil, caused by *Leishmania infantum* that were able to initially identify haematological and biochemical changes in the species, even in asymptomatic animals.

## Methods

### Study population

Ninety-eight mixed-breed equines and 26 canines of both genders were enrolled in the study. All the animals came from Uruguaiana municipality (29° 44′ 58″ S and 57° 5′ 18″ W), south of Brazil [1], which is a transmission area for VL and CVL. The collections on the individually-owned animals were carried out from November 2016 to September 2017. The subjects (equines and canines) were tested simultaneously in order to obtain a sample of animals living in the same environment, including the same owner, whenever possible. These animal samples were collected in residences of urban and semi-urban areas, whose volunteer owners used those horses as mean of transportation of people and cargo. After blood collection, the animals received an application of dewormer and were returned to their guardians.

Since the clinical signs, haematological, biochemical and oxidative stress alterations in CVL were described in other regions of Brazil, the canine specie was included to allow mean comparisons with the equine infection. The experiment started with animal’s anamnesis, the owners were asked about the place where the animals lived, the purpose of using the horses, as well as their health history, age and gender. At the time of collection, horses and dogs were submitted to general clinical evaluation to verify presence of skin and lymph node changes, body condition, hydration status, and physical examination (mucosal evaluation, capillary filling time, respiratory rate, heart rate and body temperature). Infected dogs were clinically classified as symptomatic (SyD) and asymptomatic (AD) according to the presence or absence of canine leishmaniosis signs (skin lesions, nodules, lymphadenopathy and conjunctivitis). Non-infected dogs were considered as control group (CD). The infected equines were classified with the same criteria, symptomatic equine (SyE), asymptomatic equine (AE) and non-infected group was named Control Equine (CE).

### Biological samples

The blood collection was by venepuncture of the external jugular, after carefully antisepsis, with anticoagulant EDTA (ethylene diamine tetraacetic acid) for molecular (PCR - Polymerase Chain Reaction) and haematological analyses and without anticoagulant for biochemical and oxidative stress analyses. The anticoagulant samples were frozen until the time of analyses and the samples without anticoagulant were centrifuged at 3000 rpm for 10 min and the serum frozen in aliquots.

### Molecular diagnosis

DNA from blood samples and from promastigotes of *L. (L.) infantum chagasi* (MOHM/BR/1974/PP75) was extracted using the technique by salting out [34], the volume of blood (300 μL) was standardized. The DNA purified was submitted to PCR using primers for *Leishmania infantum* that had been designed to amplify a 145 bp fragment of the conserved region of kinetoplast DNA minicircles (kDNA) [18]. Reaction mixtures were prepared in a final volume of 25 μL, containing 8 μL de DNA template, 200 μM dNTP (deoxynucleotide) (Promega, Madison, EUA), 10 pmol of each primer (IDT, Coralville, EUA), 1x buffer solution, 1.5 mM MgCl_2_, 2.5 U of Taq DNA polymerase (Invitrogen, Waltham, USA) and 8 μL of ultrapure water. The amplification conditions were as follows by Almeida et al. (2013) [18]. DNA of *L. (L.) infantum chagasi* (MOHM/BR/1974/PP75) kindly provided by *Leishmania* collection of the Oswaldo Cruz Institute (CLIOC - FIOCRUZ), was used as a positive control, while deionized water was used as a negative control for all molecular techniques. The amplicons were examined through electrophoresis on a 1.5% agarose gel stained with ethidium bromide and visualized on an AlphaImager gel documentation system with UV light.

The molecular diagnosis of infection in equine and canine samples was confirmed by direct sequencing fragments of *Leishmania* parasites, performed by an automated sequencer *ABI-Prism 3500 Genetic Analyser* (Applied Biosystems, USA), after purification of amplified PCR fragments with the *Purelink™ Quick Gel Extraction and PCR Purification kit* (Invitrogen, USA).

### Haematological, biochemical and oxidative stress analyses

The parameters evaluated in haemogram included Erythrogram: red blood cell count (RBC), haematocrit (HCT), haemoglobin (Hb) concentration and red cell indices; mean corpuscular volume (MCV), mean corpuscular haemoglobin concentration (MCHC) platelet count (PLT); Leukogram: white blood cell count (WBC), granulocytes (Neutrophils, Eosinophils, Basophils); lymphocytes, monocytes, platelet-lymphocyte ratio (PLR) and neutrophil-lymphocyte ratio (NLR). Complete blood counts were performed by an automatic cell counter (Sysmex KX-21 N, Roche®) followed by differential count and platelet count blade using the Quick Panoptic kit.

Furthermore, in vitro biochemical diagnostics were performed using an automatic biochemistry analyser (Wiener CM 200) Specific commercial kits (Bioclin - Quibasa) were used according to the manufacturers of hepatic enzymes: aspartate aminotransferase (AST) and gamma glutamyltransferase (GGT); renal enzymes: creatinine and urea; total protein, creatine kinase (CK), albumin (ALB), globulin, gamma globulin and albumin globulin ratio (A/G).

Oxidative stress markers were quantified by plasma protein carbonyl [35, 36] and plasma lipid peroxidation- thiobarbituric acid reactive substances (TBARS) [37]. All analyses were performed in duplicates.

### Statistical analyses

Animals were divided into *Leishmania infantum* infected Group and Control Group, as the results of PCR, for the statistical analyses. The values are expressed as mean ± SD for the *Leishmania infantum* infected Group and the Control Group separately. The quantitative variables were submitted to the normality test, Shapiro-Wilk test was used for canine samples and Kolmogorov-Smirnov for equine samples. Student T test was applied to normal distribution variables and Wilcoxon test was applied for non-parametric variables after transformation by Log (x + 1), both statistical tests were used to evaluate differences between independent groups. The dichotomous variables were evaluated by frequency dispersion and submitted to the Chi-square test (low body mass, skin lesions, lymphadenopathy, dehydration, yes and oral pale mucous membrane changes, gender). Data were analysed using the SPSS® for Windows computing program, significance was set at *p*-value < 0.05.

## Data Availability

The data involving in the manuscript can be obtained from the corresponding author upon reasonable request.

## Abbreviations

A/G: Albumin globulin ratio
AD: Asymptomatic dogs
AE: Asymptomatic equine
ALB: Albumin
AST: Aspartate aminotransferase
BT: Body temperature
CD: Control dogs
CE: Control equine
CEUA: Ethics Committee on Animal Use
CK: Creatine kinase
CRT: Capillary refill time
CVL: Canine visceral leishmaniosis
dNTP: Deoxynucleotide
EDTA: Ethylene diamine tetraacetic acid
GGT: Gamma glutamyltransferase
Hb: Haemoglobin
HCT: Haematocrit
HR: Heart rate
HVL: Human visceral leishmaniosis
kDNA: Kinetoplast DNA
MCHC: Mean corpuscular haemoglobin
MCV: Mean corpuscular volume
NLR: Neutrophil-lymphocyte ratio
PCR: Polymerase chain reaction
PLR: Platelet-lymphocyte ratio
PLT: Platelet
RBC: Red blood cell count
RR: Respiratory rate
SD: Standard deviations
SyD: Symptomatic dogs
SyE: Symptomatic equine
TBARS: Thiobarbituric acid reactive substances
VL: Visceral leishmaniosis
WBC: White blood cell
